# Meta-analysis of in vitro and in vivo studies of the biological effects of low-level millimetre waves

**DOI:** 10.1038/s41370-021-00307-7

**Published:** 2021-03-16

**Authors:** Andrew Wood, Rohan Mate, Ken Karipidis

**Affiliations:** 1 School of Health Sciences, Swinburne University of Technology, Melbourne, Australia; 2 Australian Radiation Protection and Nuclear Safety Agency, Melbourne, Australia

**Keywords:** Disease, Radiation, Empirical/Statistical models

## Abstract

**Background:**

With the roll-out of new technologies such as 5G, there has been renewed community concern regarding the adequacy of research on possible health effects from associated radiofrequency radiation, mainly in the millimetre wave (MMW) band.

**Objective:**

We conducted a meta-analysis of in vitro and in vivo studies investigating bioeffects of MMWs at low exposure levels.

**Methods:**

We identified 107 in vitro and in vivo studies investigating MMWs and biological effects in which the power density employed has been below 100 W/m^2^, which is below the current standards for occupational local exposures. Where possible, we estimated the magnitude of the principal effect reported or set this magnitude to zero in studies reporting no significant effects. We also estimated the quality of the studies, based on a methodology used in previous analyses.

**Results:**

We show a negative correlation between effect size and both power density and specific absorption rate. There was also a significant negative correlation between effect size and quality score. A multivariate analysis revealed that there is an increase in the effect size for certain biological systems being investigated and laboratories in which the work was carried out whilst the quality score for some of these tends to be low. We note that many of the studies were motivated by a desire to elucidate the possible mechanisms in therapeutic devices rather than assessing the safety of telecommunications systems. Finally, it appears that the presence or absence of modulation does not influence the reported effect size.

**Significance:**

Many of the findings of this meta-analysis have not been reported before and have important implications for overall interpretation of in vitro and in vivo data. Overall, the results of this study do not confirm an association between low-level MMWs and biological effects.

## Introduction

With the advent of newer telecommunications systems such as 5G and Wi-Fi 6, the spectrum above a few GHz is increasingly being used for service delivery, particularly around 26–28 GHz. There are already plans to use higher frequencies, those in the range usually termed ‘millimetre waves’ (MMW: 30–300 GHz) [1]. The International Commission for Non-Ionizing Radiation Protection (ICNIRP) has recently issued updated guidelines to cover this part of the spectrum [2] but there are suggestions that the health effects of MMW have been inadequately researched and that there could be subtle effects occurring below the ICNIRP limits, which are set largely by considering thermal effects on tissue [3]. Others have argued that the possible effects of radiofrequency (RF) exposures have been unsatisfactorily interpreted [4], advocating a delay in the 5G rollout pending further study. In a companion paper [5] we have examined 107 experimental and 31 epidemiological studies in a state-of-the-science review. The experimental studies investigated various bioeffects including genotoxicity, cell proliferation, gene expression, cell signalling, membrane function and other effects. The epidemiological studies investigated exposure to MMW from radar and a number of health effects including cancer at different sites, effects on reproduction and other diseases. The main conclusion of the companion paper was that although epidemiological studies presented little evidence of an association between MMW and any adverse health effects, the outcomes from experimental studies were more diverse, prompting a more in-depth analysis. In particular, reported bioeffects were generally not independently replicated and the majority of the studies employed low-quality methods of exposure assessment and control. This present study presents a meta-analysis of in vitro and in vivo studies investigating bioeffects of MMWs at exposure levels below the occupational level in the ICNIRP guidelines. The aim of this paper was to carry out a quantitative analysis of the papers included in the companion paper, examining in particular, questions of whether dose-response relationships can be identified, whether good quality of experimentation is associated with higher sizes of effect and whether certain themes of experiment yield higher effect sizes. The paper also aimed to investigate whether certain frequency ranges are associated with higher effect sizes.

## Methods

The search strategy for the experimental studies investigating MMWs at low exposure levels is described in the companion paper [5]. In summary, studies were found by searching the databases PubMed, EMF-Portal, Google Scholar, Embase and Web of Science using the search terms “millimeter wave”, “millimetre wave”, “gigahertz” and “GHz”. We included studies published in English where the stated RF exposure was below the occupational limit for local exposure in the ICNIRP guidelines [2]. We included only studies where there was a comparison between RF exposed and unexposed (sham), usually in an exposure chamber where the RF power was not turned on.. ‘Effect size’ is a standard statistical term for quantifying the magnitude of a phenomenon [6]. For each of the experimental studies that were included in the current analysis we estimated the effect size (ES) as the largest difference between exposed (E) and sham (S) condition (*E–S*), divided by the standard deviation (SD) of the sham condition [6], where this information could be gleaned from the information provided. In many cases, SD had to be estimated from error bars on a published figure, which involved comparing measurements on a hard copy, to give the above ratio. Where the error bars were of standard error (SE) rather than SD, SD was estimated from SE*.√n*, where *n* represented the number of observations. In some cases, *n* was not explicitly available and had to be estimated from information given in the methods or other section.

In many of the included studies more than one frequency and exposure intensity was investigated so to simplify the current analysis, the frequency at which the largest effect was observed and at the smallest exposure for a statistically significant effect was considered. In the case of studies reporting null results, the highest exposure and the main frequency of interest were considered. In some cases, several endpoints were investigated (and occasionally, more than one biological system). Here again, the largest ES was selected to represent that study. This was done to ensure that each study that was included had equal weighting in the analysis.

For each of the studies included in the analysis we followed the methodology of Vijayalaxmi and Prihoda [7] to derive a quality score (QS), using the following criteria: use of blinding; proper attention to dosimetry; use of positive controls; use of shams and in addition, temperature monitoring. For each of the studies a QS was assigned from 0 to 5 according to the number of quality criteria that were satisfied, with a 1 for the criterion to be adequately addressed, 0.5 for it to be partially addressed and 0 if absent. Two independent assessors were involved in the scoring and the resultant scores were averaged. The procedure for study selection and characterisation is summarised in Supplementary Material Fig. S1.

We have included both in vitro and in vivo experiments in this analysis. The latter category represents only 9% of the total and includes one study on human volunteers. Although the types of the study were considered separately in a multivariate analysis (see below) for most purposes they were treated as equivalent, in estimating ES and QS. The distributions of ES, power density (PD), SAR and QS were tested for normality using the Shapiro-Wilk test [8]. Because the parameters tested were not normally distributed (*p* < 0.05 for all), linear relationships between ES and PD, SAR, QS were investigated using Spearman’s rank correlation coefficient (Spearman’s ρ). A multivariate analysis was carried out to assess the variation of ES and QS according to (a) the type of study and (b) the laboratory conducting the investigation. All the analyses were performed using SPSS V. 23.

## Results

From the 107 experimental studies identified in our companion paper [5], it was possible to estimate ES according to the methodology outlined above in 88 of them. Of the remaining 19, seven were excluded because there were insufficient data to estimate SD, two related to physical rather than biological effects, two reported significant effects only for PD > 100 W/m^2^ and the others had insufficient details to evaluate the significance of the findings to the extent that would be useful in a meta-analysis of this type. The included and excluded studies are listed in Supplementary Material Table S1.

84 of the 88 studies reported PD values at or below the occupational 6-min average reference levels for local exposures, which ranges from 200 W/m^2^ at 6 GHz, dropping down to 100 W/m2 at 300 GHz [2]. Of these studies, 28 reported both specific absorption rate (SAR) and PD values, and 3 reported SAR values alone. In all but 8 studies, the reported SAR values were above 0.4 W/kg, the occupational basic restriction for this frequency range. For the general public restriction of 0.08 W/kg, all but 7 studies were above this level. However, these restrictions relate to whole-body exposures, which cannot be directly compared to in vitro values.

Papers were grouped according to the following themes (with numbers in each in brackets): bacteria and yeast (24); (non-bacterial) cells in culture (35); artificial cell suspensions (7); neural activity (10); in vivo (8) and miscellaneous (4). The purpose of grouping studies in this way was to discover whether some biological systems were more susceptible to MMW exposure than others. The miscellaneous category included five experiments on the following: human volunteers; enzyme and biomolecule activity; MMW-treated media.

Examination of the papers considered showed that some laboratories were more active than others in studying MMW bioeffects. It appears that 55% of the papers considered were from just 6 laboratories, out of a total of 30 different laboratories (Moscow Engineering Physics Institute, 11 papers; Yerevan State University, 9 papers; University of Calabria, 8 papers; Russian Academy of Sciences, Pushchino, 8 papers; University of Rennes, 7 papers; Jawaharlal Nehru University, New Delhi, 5 papers). Several studies represented a collaboration between one or more laboratories, but to simplify the analysis, the total number of separate laboratories in the analysis was kept to a minimum and each study was assigned to a single laboratory.

**Fig. 1:**
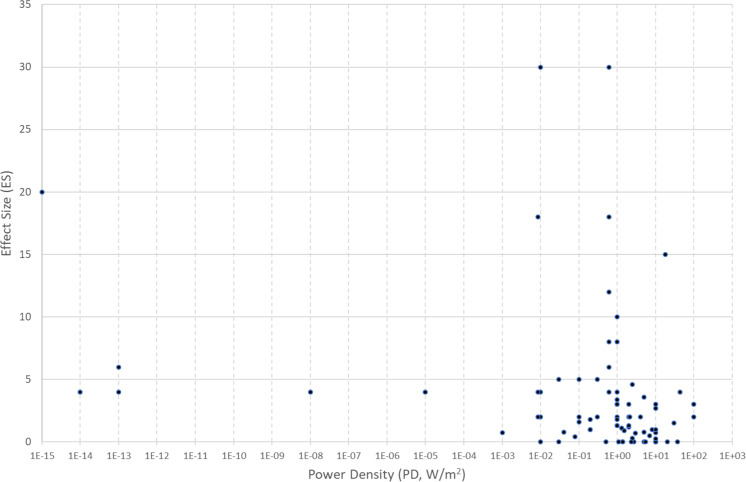
Effect size as a function of power density from 84 studies where both were reported. Effect size (ES) is defined as reported maximum difference between exposed and sham, divided by the standard deviation of sham; power density (PD) is in W/m^2^ (log scale).

**Fig. 2:**
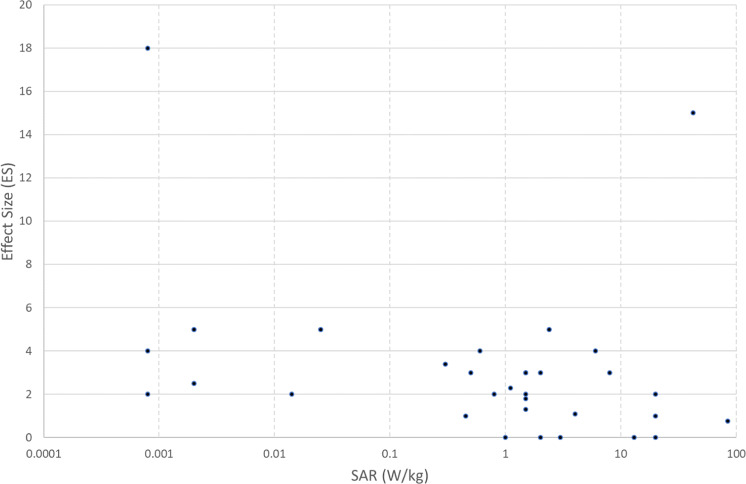
Effect size as a function of specific absorption rate in 31 studies were both were reported. Specific absorption rate (SAR) is in W/kg (log scale).

**Fig. 3:**
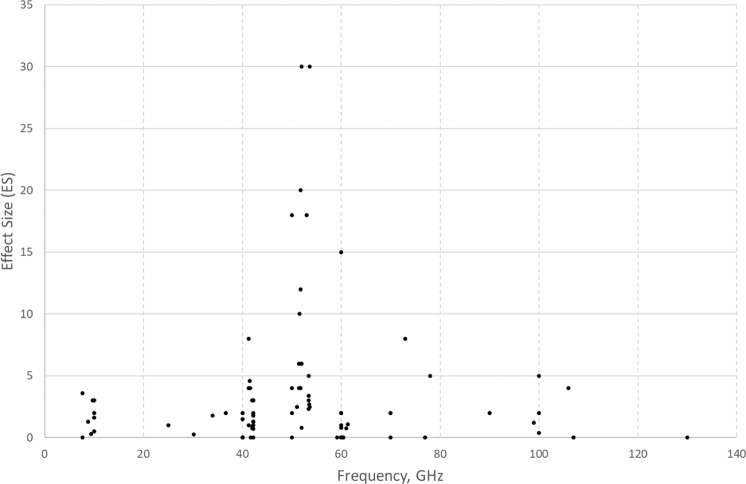
Effect size as a function of reported frequency (*n* = 88). Where multiple frequencies were investigated, the one at which the greatest ES was reported is plotted.

In Fig. 1, the values of ES are shown plotted against PD, where this was reported. This shows a large range of values from 10^–14–10+2^ W/m^2^. ES values ranged from 0 (no effect) to 30. The highest ES values appear to be associated with PD in a tight range 0.6–1 W/m^2^. However, overall there is a significant negative correlation between ES and PD values (*ρ* = −0.41, *n* = 82 *p* < 0.001). Figure 2 shows the values of ES plotted against SAR, where this was reported. The range of reported SAR values were lower (from 2 × 10^–3^ to just over 80 W/kg), and there appear to be two outlier ES values of 15 and 18 (because of the large error in estimating ES, values were rounded to the nearest integer in most cases). There appears to be a tendency for high SAR values to be associated with smaller values of ES (*ρ* = −0.37, *n* = 32, *p* < 0.05). If the two outliers are excluded, the negative correlation between ES and SAR is even greater (*ρ* = −0.44, *n* = 30, *p* < 0.02). Figure 3 shows the values of ES plotted against frequency. The most likely frequencies for large ES values to be reported were in the range 40–80 GHz, but especially 50–55 GHz. Nevertheless, there were several studies reporting null results in the same range. For the 42 studies in the latter range, there is still a negative correlation between ES and PD (*ρ* = −0.36, *n* = 37, *p* < 0.03).

**Fig. 4:**
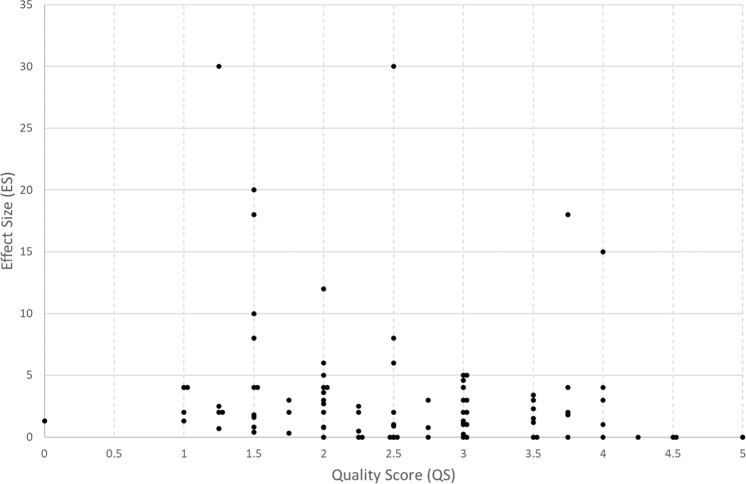
Effect size as a function of quality score. Quality score (QS) was judged by two independent scorers (scale 0–5).

Estimation of PD or SAR within the sample material requires some sophistication of analysis and the elimination of possible sources of artefact requires careful experimental design. Not all studies included in the current analysis devoted equal attention to these considerations and this is evident by the large number of studies that were assigned a low QS by the two independent assessors in our study. The degree of correlation between the two assessors was highly significant (*ρ* = 0.54, *n* = 88, *p* < 0.001). Figure 4 shows the ES as a function of QS: there is a significant negative correlation (*ρ* = −0.24, *p* < 0.03). We also considered the way the quality of studies may have changed over time. The correlation between QS and Year of Publication is highly significant (*ρ* = 0.38, *n* = 88, *p* < 0.001).

The mean values of ES were estimated according to whether the incident MMW was modulated or not. In a large proportion of studies, the type of modulation was not explicitly stated (*n* = 32). In those which stated the MMW was Continuous Wave (*n* = 36) the mean ES (±SE) was 2.2 ± 0.6, whereas those having some form of modulation (*n* = 20) the mean ES was 4.3 ± 1.6. The difference was non-significant. Unlike the variation of QS with Year of Publication, there was no such significant association between the latter and ES (*ρ* = 0.02, *n* = 88, *p* = 0.87).

A multivariate analysis was carried out to assess whether there were significant differences in the ES and QS between themes and laboratories in which the work was carried out. For themes, neural activity was chosen as the baseline theme because it had low ES and high QS values. There were significant increases in the ES for studies on bacteria and yeast (beta coefficient, *B* = 4.28, *p* < 0.001) and in vivo studies (*B* = 2.45, *p* < 0.05) compared to studies on neural activity. There was a significant decrease in the QS for studies on bacteria and yeast (beta coefficient, *B* = −0.8, *p* < 0.03).

The multivariate analysis also showed significant increases in the ES for studies conducted by certain laboratories compared to all other laboratories. Here we refer to the laboratories by an arbitrary code that we applied to them. Specifically, lab 3 (*B* = 3.33, *p* < 0.001), lab 4 (*B* = 3.4, *p* < 0.002), lab 7 (*B* = 246, *p* < 0.02) and lab 29 (*B* = 2.47, *p* < 0.05). There was a significant decrease in the QS for studies conducted by lab 3 (*B* = −1.01, *p* < 0.01) and close to a significant decrease for lab 4 (*B* = −0.62, *p* = 0.052) compared to all other laboratories.

## Discussion

### Exposure Metrics

Several studies (30) reported both SAR and PD, allowing an assessment of their ratios. This showed an average ± SE ratio of 1.75 ± 0.41 W/kg/W/m^2^, for 28 estimates (two outlying values of 600 and 142 were excluded). In adult humans, this ratio is up to 0.01 W/kg/W/m^2^ [9, 10]. The in vitro and in vivo exposure systems have in general been designed to produce efficient coupling between the MMW source and the biological material (reflected in the SAR/PD ratio). Although there is a plethora of different exposure systems, and at many different frequencies, the relatively small range may indicate that in general, the assumptions made in SAR computation could be correct. It also justifies our including the 6 studies in which the SAR was measured alone, since using this ratio would imply similar PD values to those we have considered. The high SAR to PD ratio implies that it is difficult to apply findings from in vitro or in vivo experiments to human exposure situations, since PDs at the limit values will yield SARs, in the in vitro experiments, capable of producing significant rises in temperature. In many of the experiments measures were taken to prevent temperature rise (using temperature-regulated water jackets, for example) but the possibility of ‘hot spots’ within the tissues or cell systems studied remains. The two studies with high SAR/PD ratios used modelling techniques that allowed the estimation of maximum SAR values. Since these values are the ones reported in the respective abstracts, they have been used in this analysis. In fact, we have noted a large variation in the way SAR values have been estimated and reported. Many have attempted to estimate SAR in the sample region in which the biological material is situated, but others have used less rigorous procedures. We have used reported values rather than attempting to estimate a standardised measure (such as 10 g average) but we note that there can be ratios of maximum to sample-averaged values of SAR of at least one order of magnitude. The same variability applies to reported PD values, where the point at which this has been estimated has not always been made clear. The issue of attenuation of PD in intervening layers of sample containers or media has not been addressed in most of the studies. To a certain extent, the amount of rigor involved in dosimetry estimates has been reflected in QS estimates, but this is not a fine enough measure to adequately capture the large variation in rigor or to compensate for differences in the way PD has been reported.

### Effect size

We have used Glass’s definition of ES (the difference between exposed and sham condition, divided by the SD of the sham condition) [6]. The more usual definition is to consider the pooled SD of exposed and sham conditions, but because of the variation in experimental designs, the simpler definition was adopted. There is not expected to be substantial difference between the two estimates. The estimates are in most cases rounded to the nearest integer, or for larger values to the nearest number divisible by 5. A large error attaches to ES estimates, because the data given in graphs or tables is of SE and the relevant number in the group was not always easy to discern. SE values taken from graphs assume that the graphing software used have accurately displayed the relevant data. However, because of the large number of studies considered, there should be some central tendency to allow for valid conclusions. Several of the large ES values were from studies in which a large number of end-points were considered. As mentioned, the largest effect reported in a given study formed the basis of our ES estimate. (but at the lowest exposure level giving a statistically significant change). This may bias results towards positive studies, but in dealing with a large number of very disparate studies decisions had to be made on what best characterises each study in this state-of-the-science review. This forms a caveat for our results. It may have been more appropriate to discount the estimate of SD to take account of multiple comparisons, but we chose not to do that. No distinction was drawn between the type of experiment performed (in-vitro or in vivo) and no divisions were drawn between endpoints that had an implied link to disease and those that did not. This could form the basis of further analysis.

Figure 1 is a plot of ES versus PD. There appears to be a cluster of high ES values at a PD of around 1 W/m^2^, with some anomalously high ES values at extremely low values and conversely some no effects (ES = 0) values at the highest values. Normally one would expect a positive dose-response relationship. The logarithmic trend is unexpectedly slightly downwards as mentioned above. It could be argued that there is a PD window at around 1 W/m^2^, but that seems unlikely, since there are also several studies showing no effects at this PD. The downward trend shown in Fig. 2 of ES versus log SAR is again counterfactual. Again, many of the experiments at high SAR showed no effects. It should be remarked that many of the studies which were excluded from this analysis because the PD was above 100 W/m^2^ also showed no effects, particularly those in which water jacketing or other means were employed to minimise the rise in temperature, normally expected at high SAR or PD values. It should be added that because of possible publication bias against ‘no-effects’ studies, these studies may be under-represented.

In the plot of ES versus frequency shown in Fig. 3, there appears to be a large cluster of high ES values around 40–55 GHz, but this may reflect the main frequencies available from the G4-141/2 signal generator (obtainable from Zapadpribor, Moscow), which was used in many studies. This generator is integral to a therapy device that has been used in a number of European studies over several years (and at Brooks AF Base in the US). There appear to be hardly any studies at the frequency range that will used in the next stage of the 5G roll-out, in the range 26–28 GHz. However, future mobile communications beyond the 5G network plan to use frequencies higher than 30 GHz (1) so research across the MMW band is relevant.

### Quality score

The QS value in our analysis is based on [7], but with the addition of accurate temperature measurement or temperature control. Quality rating in the context of RF studies has been used for a number of years [11] and other authors have used similar rating schemes [12]. The QS has a high error associated with it, and the ES value even more, but the plot of ES versus QS shown in Fig. 4 shows that the bulk of studies had a QS of 2 or lower, and there is a significant negative trend between ES and QS (*p* < 0.05). Only 1 study reported blinding, which is the only one to achieve 5/5. However, there are still some studies with QS of 3 or above showing clear effects, but whether these are thermal artefacts is difficult to assess. Overall, 22% show no effects and the remainder (78%) report ES ranging up to 30. The significant increase in QS with year of study would imply that greater weight should be placed on more recent studies in literature reviews, in particular, systematic reviews.

### Study themes

There are several quite distinct ‘themes’ of study, with the mean ± SD for ES as follows (numbers of studies in each theme in brackets): Bacteria and Yeast 6.4 ± 7.3 (24); Cells in culture 2.6 ± 5.5 (35); Artificial Cell suspensions: 3.1 ± 1.5 (7); Neural activity 1.4 ± 1.3 (10); in vivo: 4.3 ± 5.6 (8). The miscellaneous category had insufficient data to provide meaningful comparison. The Cells in Culture theme has the highest proportion of non-significant studies (37%). The multivariate analysis showed that studies on bacteria and yeast and in vivo studies had significant increases in the ES compared to studies on neural activity. The highest increase was for bacteria and yeast which also showed a significant decrease in the QS. Studies on cells in culture and artificial cell suspensions did not have significant increases in the ES when compared to studies on neural activity.

### Study laboratories

The multivariate analysis showed that there were significant increases in the ES for studies conducted in four particular laboratories compared to the 26 other laboratories. Two of these four showed significant decreases in the QS compared to the other laboratories. The combination of high ES with lower QS is a concern in assessing possible impacts on human health.

In many cases, the motivation to carry out the research was to find cellular evidence to back up the claimed efficacy of therapy devices, rather than the safety of telecommunication systems. Measurements of massive MIMO systems (as an indication of possible exposures following 5G roll-out) indicate that PDs at the occupational compliance boundary are below 16.1% of the occupational limits (which were 50 W/m^2^ at the time of that study) [13]. Using a value of 0.005 W/kg/W/m^2^ at these frequencies [10] implies a whole-body average of 50 × 0.161 × 0.005 = 0.04 W/kg at maximum (for the general public, the maximum values are at least 10× less). Thus, all but seven of the studies included are above the occupational compliance value of whole-body SAR. Even allowing local SAR values to be an order of magnitude greater (in whole-body exposure situations), the bulk of the literature studied is still above these SAR values and are more relevant to the study of possible therapeutic effects than detrimental effects. It should be noted that the basic restrictions for local exposure above 6 GHz are based on absorbed PD rather than SAR: at 6 GHz itself the ratio of SAR to absorbed PD [2] is 0.2 for limbs. This is based on the assumption that above this frequency the RF energy is absorbed typically in <1 mm, and this distance becomes less as frequency rises. The precise relationship between PD reported in the studies and absorbed PD is hard to evaluate, which further emphasises difficulties in extrapolating in vitro and experimental in vivo exposures to human situations.

### Implications

Although not the focus of this paper, the high SAR/PD ratio in these types of experiment make the control of temperature extremely important to incorporate into experimental design. Those studies reported here where SAR has been carefully modelled do reveal a large difference between average and maximum values within the sample volume. For example, accurate electromagnetic modelling [14] reveals a range of approximately fourfold in SAR at regions where the biological material was situated (but temperature rises were minimal, due to thermal conduction).

The new ICNIRP high-frequency guidelines pertaining to MMW exposures places limitations on absorbed PD rather than incident PD. The applicability of in vitro work is further weakened by lack of information in most cases of the PD in the layer of cells studied. Some cells are in suspension whereas others form a surface monolayer. In general, the relationship is PD = PD_0_exp(−x/λ), where λ is the absorption coefficient of the material. However, the layers may be heterogeneous, because of cuvette or Petri dish material and then subsequent media. At interfaces there will be a proportion of the incident energy reflected, so the actual PD at the layer in which the cells are located is hard to estimate. The studies involving exposure of live animals have a different relationship between the incident and absorbed PD. This further emphasises the care which should be taken in extrapolating in vitro or in vivo results to assessing implications for human health.

It has been suggested by several commentators that the presence of modulation is the critical factor on whether RF radiation can exert a low-level bioeffect or not [3, 4]. However, the results of this analysis show no significant difference in average ES between those studies in which modulation was used and those without.

Data from studies of the effects of MMW below the ICNIRP PD limits should be used with caution, because the estimated SAR values could be several orders of magnitude above the corresponding ICNIRP limits. It is also important to be aware that in many cases the rationale for undertaking such studies has been to elucidate the mechanisms of interaction underlying the reported efficacy of MMW therapy devices, especially in the frequency range 40–75 GHz. Nevertheless, although the tendency is for higher PD or higher QS to be associated with smaller ES values, the persistence of non-zero ES values implies either that unknown artefacts are in play or that genuine effects are occurring at levels below the occupational limits but where the precise conditions for consistently observing such effects are at present elusive. However, the weight of evidence appears to be that such effects have not been unequivocally demonstrated.

## Conclusions

This meta-analysis showed that there was no dose-response relationship between the exposure (either PD or SAR) and the ES. In fact, studies with a higher exposure counterfactually show a lower ES. Most of the studies showing a large ES were conducted in the frequency range around 40–55 GHz, representing investigations into the use of MMWs for therapeutic purposes, rather than deleterious health consequences. However, the counterfactual inverse relationship between PD and ES is still significant for this range. Future experimental research would benefit from investigating bioeffects at the specific frequency range of the next stage of the 5 G network roll-out in the range 26–28 GHz and higher designated bands above 30 GHz. The estimated quality of studies has increased in recent years, so more weight should be attached to the more recent studies. Many of the findings of this meta-analysis have not been reported before and have important implications for overall interpretation of in vitro and in vivo data. Nevertheless, there is little consistent evidence to support the notion of biological effects from MMWs at levels below the ICNIRP occupational limits. Since general public limits are ten times lower, it appears that the evidence for effects at these levels is even less.

## Supplementary Information

Figure S1

Table S1
