# Supplementary material for: Meta-analysis of in vitro and in vivo studies of the biological effects of low-level millimetre waves
Source: J Expo Sci Environ Epidemiol. 2021 Mar 16;31(4):606–13. doi: 10.1038/s41370-021-00307-7 (PMC7962924; doi:10.1038/s41370-021-00307-7)

Figure S1. Flow diagram of study identification, extraction of exposure parameters, effect size estimation and quality score process.

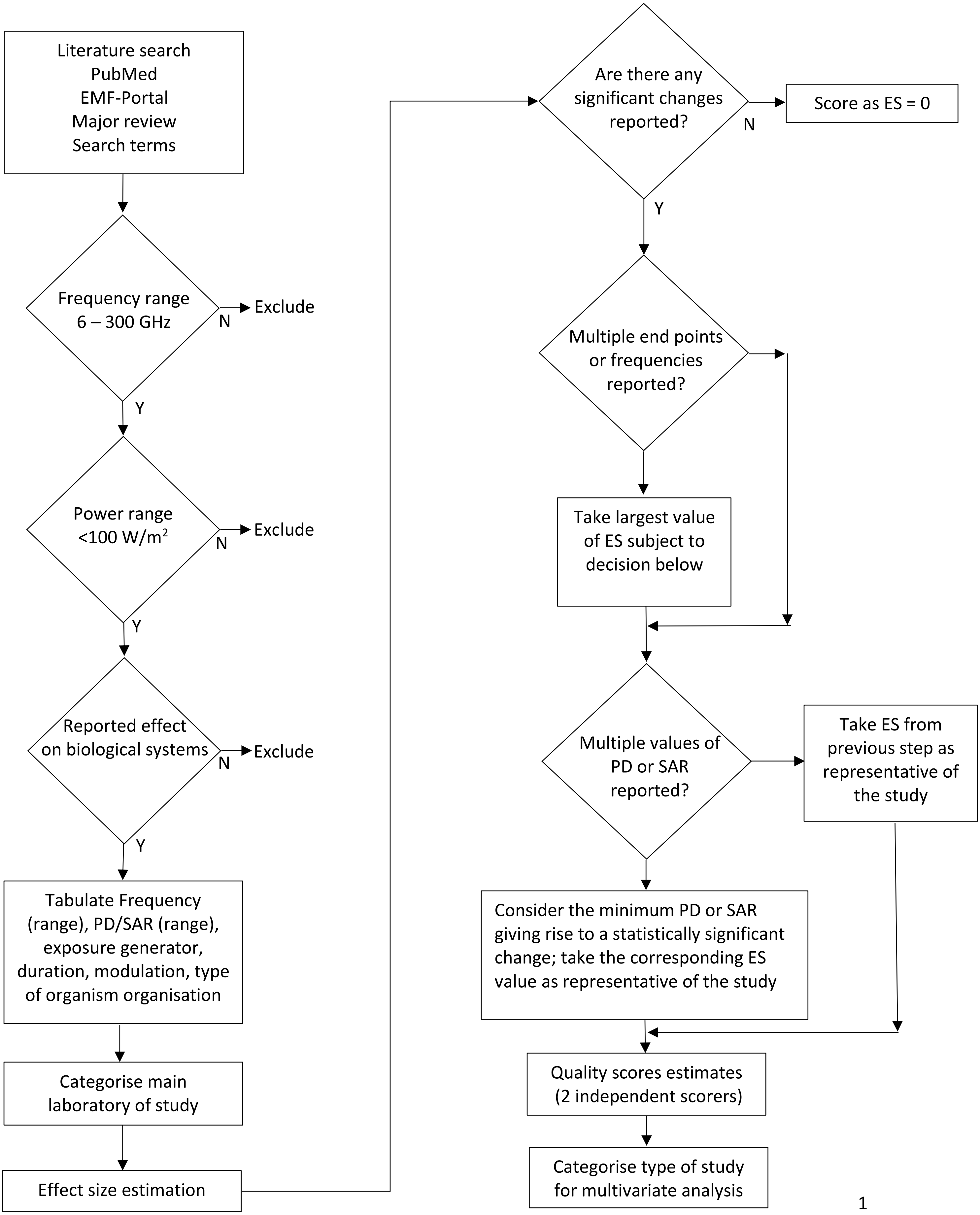

Supplement: Supplementary file 1 — Figure S1 [file 41370_2021_307_MOESM1_ESM.pdf]
