# Supplementary material for: Meta-analysis of in vitro and in vivo studies of the biological effects of low-level millimetre waves
Source: J Expo Sci Environ Epidemiol. 2021 Mar 16;31(4):606–13. doi: 10.1038/s41370-021-00307-7 (PMC7962924; doi:10.1038/s41370-021-00307-7)
Supplement: Supplementary file 2 — Table S1 [file 41370_2021_307_MOESM2_ESM.pdf]

Table S1. List of studies included in meta-analysis (n = 88). Of the 107 papers considered in the companion paper to this one, 19 were excluded, for reasons summarised in this paper. These studies are listed at the end of this Table.

Authors, year	*Country*	*Frequency (GHz)*	*Intensity (W/m²)*	*SAR (W/kg)*	*Effect size*
Badzhinyan et al (2000) [1]	*Armenia*	*40*	*100*		*2*
Bellossi et al (2000) [2]	*Japan*	*60*	*5*		*0.8*
Belyaev and Kravchenko (1994) [3]	*Russia*	*41.6*	*1.00E-05*		*4*
Belyaev et al (1992) [4]	*Russia*	*51.7*	*0.01*	*6*	*4*
Belyaev et al (1992) [5]	*Russia*	*51.7*	*1*	*0.6*	*4*
Belyaev et al (1992) [6]	*Russia*	*51.6*	*1*		*10*
Belyaev et al (1993) [7]	*Russia*	*41.3*	*1.00E-08*		*4*
Belyaev et al (1993) [8]	*Russia*	*41.3*	*1*		*8*
Belyaev et al (1994) [9]	*Russia*	*51.8*	*1.00E-15*		*20*
Belyaev et al (1996) [10]	*Russia*	*51.7*	*1.00E-14*		*4*
Beneduci (2009) [11]	*Italy*	*42.2*	*1.1*		*0*
Beneduci et al (2005) [12]	*Italy*	*51.1*			*2.5*
Beneduci et al (2007) [13]	*Italy*	*53.6*	*0.01*		*30*
Beneduci et al (2012) [14]	*Italy*	*53.6*		*0.002*	*2.5*
Beneduci et al (2013) [15]	*Italy*	*53.4*	*0.1*	*0.002*	*5*
Beneduci et al (2014) [16]	*Italy*	*78*	*0.03*	*0.025*	*5*
Chen et al (2004) [17]	*China*	*30.2*	*10*		*0.25*
Cohen et al (2009) [18]	*Israel*	*99*	*2.00E+00*	*283*	*1.2*
Cosentino et al (2013) [19]	*Italy*	*52*	*0.04*		*0.8*
Crouzier et al (2009) [20]	*India*	*9.71*		*8*	*3*
D'Agostino et al (2018) [21]	*Italy*	*53.37*		*1.1*	*2.3*
De Amicis et al (2015) [22]	*Italy*	*100*	*4*	*20*	*2*
Deghoyan et al (2012) [23]	*Armenia*	*90*		*1.5*	*2*
Di Donato et al (2012) [24]	*Italy*	*53.37*	*1*	*0.3*	*3.4*
Franchini et al (2018) [25]	*Italy*	*25*	*8*	*20*	*1*
Furia et al (1986) [26]	*US*	*41.7*			*0*
Gandhi et al (1980) [27]	*US*	*50*	*37*		*0*
Gapeyev and Lukyanova (2015) [28]	*Russia*	*42.2*	*1*	*1.50E+00*	*1.8*
Gapeyev et al (1997) [29]	*Russia*	*42*	*1.00E-03*		*0.75*
Gapeyev et al (1998) [30]	*Russia*	*42*	*1.5*		*0.9*
Gapeyev et al (2011) [31]	*Russia*	*42.2*	*1*	*1.5*	*3*
Gapeyev et al (2013) [32]	*Russia*	*42.2*	*1*	*1.5*	*3*
Gapeyev et al (2014) [33]	*Russia*	*42.2*	*1*	*1.5*	*1.3*
Garaj-Vrhovac et al (1991) [34]	*Croatia*	*7.7*	*5*		*3.6*
Garaj-Vrhovac et al (1992) [35]	*Croatia*	*7.7*	*5*		*0*

Geletyuk et al (1995) [36]	*Russia*	*42.25*	*1*		*1.3*
Gos et al (1997) [37]	*Switzerland*	*40*	*0.5*	*1*	*0*
Grundler and Keilmann (1983) [38]	*France*	*42*	*1.00E+02*		*3*
Hintzsche et al (2011) [39]	*Germany*	*106*	*43*		*4*
Hintzsche et al (2012) [40]	*Germany*	*107*	*20*	*13*	*0*
Homenko et al (2009) [41]	*Israel*	*100*	*0.08*		*0.4*
Hovnanyan et al (2017) [42]	*Armenia*	*51.8*	*0.6*		*12*
Kesari and Behari (2010) [43]	*India*	*50*	*0.0086*	*8.00E-04*	*2*
Kesari and Behari (2009) [44]	*India*	*50*	*0.0086*	*8.00E-04*	*4*
Korenstein-Ilan et al (2008) [45]	*Israel*	*100*	*0.3*	*2.4*	*5*
Koyama et al (2016) [46]	*Japan*	*60*	*10*		*0*
Koyama et al (2019) [47]	*Japan*	*40*	*10*		*0*
Kumar et al (2010) [48]	*India*	*50*	*0.0086*	*8.00E-04*	*18*
Kumar et al (2011) [49]	*India*	*10*	*2.1*	*0.014*	*2*
Le Quement et al (2012) [50]	*France*	*60*	*18*	*4.24E+01*	*15*
Lukashevsky and Belyaev (1990) [51]	*Russia*	*70*	*1*	*0.8*	*2*
Minasyan et al (2007) [52]	*Armenia*	*42.2*	*3*		*0.7*
Muller et al (2004) [53]	*Germany*	*77*	*0.03*		*0*
Munemori and Ikeda (1982) [54]	*Japan*	*9.4*	*2.5*		*0.3*
Munemori and Ikeda (1984) [55]	*Japan*	*10*	*7*		*0.5*
Nicolaz et al (2008) [56]	*France*	*60.4*	*1.4*		*0*
Nicolaz et al (2009) [57]	*France*	*59.2*	*1.4*	*3*	*0*
Olchowik and Maj (2000) [58]	*Poland*	*53.57*	*10*		*2.7*
Pakhomova et al (1997) [59]	*US*	*61.3*	*1.3*	*4*	*1.1*
Pakhomov et al (1997) [60]	*US*	*40*	*30*		*1.5*
Pakhomov et al, (1997) [61]	*US*	*41.5*	*2.5*		*4.6*
Pakhomov et al (1997) [62]	*US*	*41.3*	*0.2*		*1*
Paulraj and Behari (2006) [63]	*India*	*10*	*10*	*2*	*3*
Pikov et al (2010) [64]	*US*	*60*	*0.01*		*2*
Pikov and Siegel (2011) [65]	*US*	*60*	*2*		*2*
Ramundo-Orlando et al (2009) [66]	*Italy*	*53.37*	*1*	*0.5*	*3*
Rojavin and Ziskin (1995) [67]	*US*	*61*	*10*	*84*	*0.75*
Romanenko et al (2014) [68]	*US*	*60*	*10*		*1*

Rotkovská et al (1993) [69]	*Czech Rep*	*34*	*0.2*		*1.8*
Safronova et al (2002) [70]	*Russia*	*42.2*	*0.2*	*0.45*	*1*
Sarapultseva et al (2014) [71]	*Russia*	*10*	*0.1*		*1.6*
Shcheglov et al (1997) [72]	*Russia*	*51.5*	*1.00E-13*		*4*
Shcheglov et al (2002) [73]	*Russia*	*51.5*	*1.00E-13*		*6*
Shckorbatov et al (1998) [74]	*Ukraine*	*42*	*2*		*3*
Shckorbatov et al (2002) [75]	*Ukraine*	*8.75*	*2*		*1.3*
Shckorbatov et al (2010) [76]	*Ukraine*	*36.7*	*0.1*		*2*
Shiina et al (2014) [77]	*Japan*	*60*	*10*		*0*
Soghomonyan and Trchounian (2013) [78]	*Armenia*	*51.8*	*0.6*		*4*
Tadevosyan et al (2008) [79]	*Armenia*	*52*	*0.6*		*6*
Torgomyan (2011) [80]	*Armenia*	*73*	*0.6*		*8*
Torgomyan et al (2012) [81]	*Armenia*	*52*	*0.6*		*30*
Torgomyan et al (2012) [82]	*Armenia*	*53*	*0.6*		*18*
Volkova et al (2014) [83]	*Ukraine*	*42.25*	*0.3*		*2*
Yaekashiwa et al (2017) [84]	*Japan*	*70*	*0.01*		*0*
Zeni et al (2007) [85]	*Italy*	*130*	*2.3*	*2*	*0*
Zhadobov et al (2005) [86]	*France*	*60*	*2.7*		*0*
Zhadobov et al (2007) [87]	*France*	*60*	*5.4*		*0*
Zhadobov et al (2009) [88]	*France*	*60.42*	*10*	*20*	*0*

References of included studies

1. Badzhinyan, SA, Sayadyan, AB, Sarkisyan, NK, Grigoryan, R.M. and Gasparyan, GG. Lethal effect of electromagnetic radiation of the millimeter wavelength range on cell cultures of chicken embryo. Dokl Biochem Biophys 2001; 377:94-5.
2. Bellossi A, Dubost G, Moulinoux JP, Himdi M, Ruelloux M, Rocher C. Biological effects of millimeter wave irradiation on mice-preliminary results**.** IEEE Trans on Microw Theory Tech 2000; 48(11):2104-2110.
3. Belyaev IY, Kravchenko VG. Resonance effect of low-intensity millimeter waves on the chromatin conformational state of rat thymocytes. Z Naturforsch 1994; 49(5-6):352-8.
4. Belyaev IY, Alipov YD, Shcheglov VS, Lystsov VN. Resonance effect of microwaves on the genome conformational state of E. coli cells. Z NaturforschC 1992; 47(7-8):621-7.

5. Belyaev I, Alipov YD, Shcheglov VS. 1992. Chromosome DNA as a target of resonant interaction between Escherichia coli cells and low–intensity millimeter waves. Electro Magnetobiol 11(2):97-108.
6. Belyaev IY, Shcheglov VS, Alipov YD. Selection rules on helicity during discrete transitions of the genome conformational state in intact and X-rayed cells of E. coli in millimeter range of electromagnetic field. Charge and Field Effects in Biosystems 1992; 3:115-126.
7. Belyaev IY, Alipov YD, Polunin VA, Shcheglov VS. Evidence for dependence of resonant frequency of millimeter wave interaction with Escherichia coli K12 cells on haploid genome length. Electro Magnetobiol 1993; 12(1):39-49.
8. Belyaev IY, Shcheglov VS, Alipov YD, Radko SP. Regularities of separate and combined effects of circularly polarized millimeter waves on E. coli cells at different phases of culture growth. Bioelectrochem Bioenerg 1993; 31(1):49-63.
9. Belyaev IY, Alipov YD, Shcheglov VS, Polunin VA, Aizenberg OA. Cooperative response of Escherichia coli cells to the resonance effect of millimeter waves at super low intensity. Electro Magnetobiol 1994; 13(1):53-66.
10. Belyaev IY, Shcheglov VS, Alipov YD, Polunin VA. Resonance effect of millimeter waves in the power range from 10-19 to 3× 10-3 W/cm2 on Escherichia coli cells at different concentrations. Bioelectromagnetics 1996; 17(4):312-21.
11. Beneduci A. Evaluation of the potential in vitro antiproliferative effects of millimeter waves at some therapeutic frequencies on RPMI 7932 human skin malignant melanoma cells. Cell Biochem Biophys 2009; 1;55(1):25-32.
12. Beneduci A, Chidichimo G, Tripepi S, Perrotte E. Frequency and Irradiation Time-dependant Antiproliferative Effect of Low-power Millimeter Waves on RPMI 7932 Human Melanoma Cell Line. Anticancer Res 2005; 25(2A):1023-1028.
13. Beneduci A, Chidichimo G, Tripepi S, Perrotta E, Cufone F. Antiproliferative effect of millimeter radiation on human erythromyeloid leukemia cell line K562 in culture: Ultrastructural-and metabolic-induced changes. Bioelectrochemistry 2007; 70(2):214-20.
14. Beneduci A, Filippelli L, Cosentino K, Calabrese ML, Massa R, Chidichimo G. 2012. Microwave induced shift of the main phase transition in phosphatidylcholine membranes. Bioelectrochemistry 2012; 1;84:18-24.
15. Beneduci A, Cosentino K, Chidichimo G. Millimeter wave radiations affect membrane hydration in phosphatidylcholine vesicles. Materials 2013; 6(7):2701-12.
16. Beneduci A, Cosentino K, Romeo S, Massa R, Chidichimo G. Effect of millimetre waves on phosphatidylcholine membrane models: a non-thermal mechanism of interaction. Soft Matter 2014; 10(30):5559-67.
17. Chen Q, Zeng QL, Lu DQ, Chiang H. Millimeter wave exposure reverses TPA suppression of gap junction intercellular communication in HaCaT human keratinocytes. Bioelectromagnetics 2004; 25(1):1-4.
18. Cohen I, Cahan R, Shani G, Cohen E, Abramovich A. Effect of 99 GHz continuous millimeter wave electro-magnetic radiation on E. coli viability and metabolic activity. Int J Radiat Biol 2010; 86(5):390-9.
19. Cosentino K, Beneduci A, Ramundo-Orlando A, Chidichimo G. The influence of millimeter waves on the physical properties of large and giant unilamellar vesicles. J Biol Phys 2013; 39(3):395-410.

20. Crouzier D, Perrin A, Torres G, Dabouis V, Debouzy JC. Pulsed electromagnetic field at 9.71 GHz increase free radical production in yeast (Saccharomyces cerevisiae). Patho Biol 2009; 57(3):245-251.

21. D'Agostino S, Della Monica C, Palizzi E, Di Pietrantonio F, Benetti M, Cannatà D et al. Extremely high frequency electromagnetic fields facilitate electrical signal propagation by increasing transmembrane potassium efflux in an artificial axon model. Scientific Reports 2018; 8(1):9299.

22. De Amicis A, Sanctis SD, Cristofaro SD et al. Biological effects of *in vitro* THz radiation exposure in human foetal fibroblasts. Mutat Res Genet Toxicol Environ Mutagen 2015; 793:150–60.

23. Deghoyan A, Heqimyan A, Nikoghosyan A, Dadasyan E, Ayrapetyan S. Cell bathing medium as a target for non thermal effect of millimeter waves. Electromag Biol Med 2012; 31(2):132-42.

24. Di Donato L, Cataldo M, Stano P, Massa R, Ramundo-Orlando A. Permeability changes of cationic liposomes loaded with carbonic anhydrase induced by millimeter waves radiation. Radiat Res 2012; 178(5):437-46.

25. Franchini V, Regalbuto E, De Amicis A, De Sanctis S, Di Cristofaro S, Coluzzi E et al. Genotoxic effects in human fibroblasts exposed to microwave radiation. Health Phys; 2018 115(1):126-39.

26. Furia L, Hill DW, Gandhi OMP. Effect of millimeter-wave irradiation on growth of Saccharomyces cerevisiae. IEEE Trans Biom Eng 1986; (11):993-9.

27. Gandhi OP, Hagmann MJ, Hill DW, Partlow LM, Bush L. Millimeter wave absorption spectra of biological samples. Bioelectromagnetics 1980; 1(3):285-98.

28. Gapeyev AB, Lukyanova NA. Pulse-modulated extremely high-frequency electromagnetic radiation protects cellular DNA from the damaging effects of physical and chemical factors in vitro. Biophys 2015; 60(5):732-738.

29. Gapeyev AB, Safronova VG, Chemeris NK, Fesenko EE. Inhibition of the production of reactive oxygen species in mouse peritoneal neutrophils by millimeter wave radiation in the near and far field zones of the radiator. Bioelectrochemand Bioenerg 1997; 43(2):217-20.

30. Gapeyev AB, Yakushina VS, Chemeris NK, Fesenko EE. Modification of production of reactive oxygen species in mouse peritoneal neutrophils on exposure to low-intensity modulated millimeter wave radiation. Bioelectrochem Bioenerg 1998; 46(2):267-72.

31. Gapeyev AB, Kulagina TP, Aripovsky AV, Chemeris NK. The role of fatty acids in anti-inflammatory effects of low-intensity extremely high-frequency electromagnetic radiation. Bioelectromagnetics 2011; 32(5):388-95.

32. Gapeyev AB, Kulagina TP, Aripovsky AV. Exposure of tumor-bearing mice to extremely high-frequency electromagnetic radiation modifies the composition of fatty acids in thymocytes and tumor tissue. Int J Radiat Biol 2013; 89(8):602-10.

33. Gapeyev A, Lukyanova N, Gudkov S. Hydrogen peroxide induced by modulated electromagnetic radiation protects the cells from DNA damage. Open Life Sci 2014; 9(10):915-21.

34. Garaj-Vrhovac V, Horvat D, Koren Z. The relationship between colony-forming ability, chromosome aberrations and incidence of micronuclei in V79 Chinese hamster cells exposed to microwave radiation. Mutat Res Letters 1991; 263(3):143-9.
35. Garaj-Vrhovac V, Fučić A, Horvat D. The correlation between the frequency of micronuclei and specific chromosome aberrations in human lymphocytes exposed to microwave radiation in vitro. Mutat Res Letters 1992; 281(3):181-6.
36. Geletyuk VI, Kazachenko VN, Chemeris NK, Fesenko EE. Dual effects of microwaves on single Ca2+-activated K+ channels in cultured kidney cells Vero. FEBS letters 1995; 359(1):85-8.
37. Gos P, Eicher B, Kohli J, Heyer WD. Extremely high frequency electromagnetic fields at low power density do not affect the division of exponential phase Saccharomyces cerevisiae cells. Bioelectromagnetics 1997; 18(2):142-55.
38. Grundler, W. and Keilmann, F. Sharp resonances in yeast growth prove nonthermal sensitivity to microwaves. Phys Rev letters 1983; 51(13): 1214.
39. Hintzsche H, Jastrow C, Kleine-Ostmann T. Terahertz radiation induces spindle disturbances in human-hamster hybrid cells. Radiat Res 2011; 175:569–74.
40. Hintzsche H, Jastrow C, Kleine-Ostmann T, Kärst U, Schrader T, Stopper H. Terahertz electromagnetic fields (0.106 THz) do not induce manifest genomic damage *in vitro*. PloS One 2012; 7(9).
41. Homenko A, Kapilevich B, Kornstein R, Firer MA. Effects of 100 GHz radiation on alkaline phosphatase activity and antigen–antibody interaction. Bioelectromagnetics 2009; 30(3):167-75.
42. Hovnanyan K, Kalantaryan V, Trchounian A. The distinguishing effects of low-intensity electromagnetic radiation of different extremely high frequencies on Enterococcus hirae: growth rate inhibition and scanning electron microscopy analysis. Letters in Appl microbiol 2017; 65(3):220-5.
43. Kesari KK, Behari J. Microwave exposure affecting reproductive system in male rats. Appl biochem and biotechnol 2010; 162(2):416-428.
44. Kesari KK, Behari J. Fifty-gigahertz microwave exposure effect of radiations on rat brain. Appl biochem and biotechnol 2009; 158(1):126.
45. Korenstein-Ilan A, Barbul A, Hasin P et al. Terahertz radiation increases genomic instability in human lymphocytes. Radiat Res 2008; 170:224–34.
46. Koyama S, Narita E, Shimizu Y, Suzuki Y, Shiina T, Taki M, et al. Effects of long-term exposure to 60 GHz millimeter-wavelength radiation on the genotoxicity and heat shock protein (Hsp) expression of cells derived from human eye. Int J Environ Res public health 2016; 13(8):802.
47. Koyama S, Narita E, Suzuki Y, Shiina T, Taki M, Shinohara N et al. Long-term exposure to a 40-GHz electromagnetic field does not affect genotoxicity or heat shock protein expression in HCE-T or SRA01/04 cells. J Radiat Res 2019; 60(4):417-23.

48. Kumar S, Kesari KK, Behari J. Evaluation of genotoxic effects in male Wistar rats following microwave exposure. Indian J Exp Biol 2010; 48(6):586-92.

49. Kumar S, Kesari KK, Behari J. Influence of microwave exposure on fertility of male rats. Fertility and sterility 2011; 95(4):1500-1502.

50. Le Quément C, Nicolas Nicolaz C, Zhadobov M, Desmots F, Sauleau R, Aubry M et al. Whole-genome expression analysis in primary human keratinocyte cell cultures exposed to 60 GHz radiation. Bioelectromagnetics 2012; 33(2):147-158.

51. Lukashevsky KV, Belyaev IY. Switching of prophage lambda genes in Escherichia coli by millimetre waves. Med Sci Res 1990; 18(24):955-7.

52. Minasyan SM, Grigoryan GY, Saakyan SG, Akhumyan AA, Kalantaryan VP. Effects of the action of microwave-frequency electromagnetic radiation on the spike activity of neurons in the supraoptic nucleus of the hypothalamus in rats. Neurosci Behav Physiol 2007; 37(2):175-80.

53. Müller J, Hadeler KP, Müller V, Waldmann J, Landstorfer FM, Wisniewski R et al. Influence of low power cm-/mm-microwaves on cardiovascular function. Int J Environ Health Res 2004; 14(5):331-41.

54. Munemori J, Ikeda T. Effects of low-level microwave radiation on the eye of the crayfish. Med Biol Eng Compu 1982; 20(1):84-8.

55. Munemori J, Ikeda T. Biological effects of X-band microwave radiation on the eye of the crayfish. Med Biol Eng Compu 1984; 22(3):263-7.

56. Nicolaz CN, Zhadobov M, Desmots F, Ansart A, Sauleau R, Thouroude D, et al. Study of narrow band millimeter-wave potential interactions with endoplasmic reticulum stress sensor genes. Bioelectromagnetics 2008; 30(5):365-73.

57. Nicolaz CN, Zhadobov M, Desmots F, Sauleau R, Thouroude D, Michel D et al. Absence of direct effect of low-power millimeter-wave radiation at 60.4 GHz on endoplasmic reticulum stress. Cell Biol Toxicol 2009; 25(5):471-8.

58. Olchowik G, Maj JG. Inhibitory action of microwave radiation on gamma-glutamyl transpeptidase activity in liver of rats treated with hydrocortisone. Folia Histochemica Et Cytobiologica 2000; 38(4):189-91.

59. Pakhomova ON, Pakhomov AG, Akyel Y. Effect of millimeter waves on UV-induced recombination and mutagenesis in yeast. Bioelectrochem Bioenerg 1997; 43(2):227-32.

60. Pakhomov AG, Prol HK, Mathur SP, Akyel Y, Campbell CB. Frequency-specific effects of millimeter-wavelength electromagnetic radiation in isolated nerve. Electro Magnetobiol 1997; 16(1):43-57.

61. Pakhomov AG, Prol HK, Mathur SP, Akyel Y, Campbell CB. Search for frequency-specific effects of millimeter-wave radiation on isolated nerve function. Bioelectromagnetics 1997; 18(4):324-34.

62. Pakhomov AG, Prol HK, Mathur SP, Akyel Y, Campbell CB. Role of field intensity in the biological effectiveness of millimeter waves at a resonance frequency. Bioelectrochem Bioenerg 1997; 43(1):27-33.

63. Paulraj R, Behari J. Single strand DNA breaks in rat brain cells exposed to microwave radiation. Mutat Res 2006; 596(1-2):76-80.

64. Pikov V, Arakaki X, Harrington M, Fraser SE, Siegel PH. Modulation of neuronal activity and plasma membrane properties with low-power millimeter waves in organotypic cortical slices. J Neural Eng 2010; 7(4):045003.

65. Pikov V, Siegel PH. Millimeter wave-induced changes in membrane properties of leech Retzius neurons. Photonic Therapeutics and Diagnostics 2011; 7883:56-1

66. Ramundo-Orlando A, Longo G, Cappelli M, Girasole M, Tarricone L, Beneduci A et al. The response of giant phospholipid vesicles to millimeter waves radiation. Biochem Biophys Acta 2009; 1788(7):1497-1507.

67. Rojavin MA, Ziskin MC. Effect of millimeter waves on survival of UVC-exposed Escherichia coli. Bioelectromagnetics 1995; 16(3):188-96.

68. Romanenko S, Siegel PH, Wagenaar DA, Pikov V. Effects of millimeter wave irradiation and equivalent thermal heating on the activity of individual neurons in the leech ganglion. J Neurophysiol 2014; 112(10):2423-31.

69. Rotkovská D, Moc J, Kautská J, Bartonícková A, Keprtová J, Hofer M. Evaluation of the biological effects of police radar RAMER 7F. Environ Health Perspect 1993; 101(2):134-6.

70. Safronova VG, Gabdoulkhakova AG, Santalov BF. Immunomodulating action of low intensity millimeter waves on primed neutrophils. Bioelectromagnetics 2002; 23(8):599-606.

71. Sarapultseva EI, Igolkina JV, Tikhonov VN, Dubrova YE. The in vivo effects of low-intensity radiofrequency fields on the motor activity of protozoa. Int J Radiat Biol 2014; 90(3):262-7.

72. Shcheglov VS, Belyaev I, Alipov YD, Ushakov VL. Power-dependent rearrangement in the spectrum of resonance effect of millimeter waves on the genome conformational state of Escherichia Coli cells. Electro Magnetobiol 1997; 16(1):69-82.

73. Shcheglov VS, Alipov ED, Belyaev I. Cell-to-cell communication in response of E. coli cells at different phases of growth to low-intensity microwaves. Biochimica et biophysica acta (BBA)-general subjects 2002; 1572(1):101-6.

74. Shckorbatov YG, Grigoryeva NN, Shakhbazov VG, Grabina VA, Bogoslavsky AM. Microwave irradiation influences on the state of human cell nuclei. Bioelectromagnetics; 1998 19(7):414-9.

75. Shckorbatov YG, Shakhbazov VG, Navrotskaya VV, Grabina VA, Sirenko SP, Fisun AI et al. Application of intracellular microelectrophoresis to analysis of the influence of the low-level microwave radiation on electrokinetic properties of nuclei in human epithelial cells. Electrophoresis 2002; 23(13):2074-2079.

76. Shckorbatov YG, Pasiuga VN, Goncharuk EI, Petrenko TP, Grabina VA, Kolchigin NN et al. Effects of differently polarized microwave radiation on the microscopic structure of the nuclei in human fibroblasts. J Zhejiang Unive Sci B 2010; 11(10):801-805.

77. Shiina T, Suzuki Y, Kasai Y, Inami Y, Taki M, Wake K. Effect of two-times 24 hour exposures to 60 GHz millimeter-waves on neurite outgrowth in PC12VG cells in consideration of polarization. 2014. IEEE Int Sympo Electromag Compat 2014; 166-169.

78. Soghomonyan D, Trchounian A. Comparable effects of low-intensity electromagnetic irradiation at the frequency of 51.8 and 53 GHz and antibiotic ceftazidime on Lactobacillus acidophilus growth and survival. Cell Biochem Biophys 2013; 67(3):829-35.

79. Tadevosyan H, Kalantaryan V, Trchounian A. Extremely high frequency electromagnetic radiation enforces bacterial effects of inhibitors and antibiotics. Cell Biochem Biophys 2008; 51(2-3):97-103.

80. Torgomyan H, Kalantaryan V, Trchounian A. Low intensity electromagnetic irradiation with 70.6 and 73 GHz frequencies affects Escherichia coli growth and changes water properties. Cell Biochem Biophys 2011; 60(3):275-81.

81. Torgomyan H, Hovnanyan K, Trchounian A. Escherichia coli growth changes by the mediated effects after low-intensity electromagnetic irradiation of extremely high frequencies. Cell Biochem Biophys 2012; 65(3):445-54.

82. Torgomyan H, Ohanyan V, Blbulyan S, Kalantaryan V, Trchounian A. Electromagnetic irradiation of Enterococcus hirae at low-intensity 51.8-and 53.0-GHz frequencies: changes in bacterial cell membrane properties and enhanced antibiotics effects. FEMS microbiol letters 2012; 329(2):131-7.

83. Volkova NA, Pavlovich EV, Gapon AA, Nikolov OT. Effects of millimeter-wave electromagnetic exposure on the morphology and function of human cryopreserved spermatozoa. Bull Exp Biol Med 2014; 157(5):574-6.

84. Yaekashiwa N, Otsuki S, Hayashi SI, Kawase K. 2017. Investigation of the non-thermal effects of exposing cells to 70–300 GHz irradiation using a widely tunable source. J Radiat Res 59(2):116-21.

85. Zeni O, Gallerano GP, Perrotta A, Romano M, Sannino A, Sarti M et al. Cytogenetic observations in human peripheral blood leukocytes following in vitro exposure to THz radiation: a pilot study. Health Phys 2007; 92(4):349-57.

86. Zhadobov M, Sauleau R, Le Coq L, Thouroude D, Orlov I, Michel D et al. 60 GHz electromagnetic fields do not activate stress-sensitive gene expression. IEEE 11th Int Sympo on Antenna Technol and appl electromag 2005; 1-4.

87. Zhadobov M, Sauleau R, Le Coq L, Debure L, Thouroude D, Michel D et al. Low-power millimeter wave radiations do not alter stress-sensitive gene expression of chaperone proteins. Bioelectromagnetics 2007; 28(3):188-96.

88. Zhadobov M, Nicolaz CN, Sauleau R, Desmots F, Thouroude D, Michel D et al. Evaluation of the potential biological effects of the 60-GHz millimeter waves upon human cells. IEEE Trans Antennas Propag 2009; 57(10):2949-56.

References of excluded studies

The reasons for exclusion of these references is summarised in the main paper.

1. Belyaev IY, Shcheglov VS, Alipov YD. Existence of selection rules on helicity during discrete transitions of the genome conformational state of E. coli cells exposed to low-level millimetre radiation. Bioelectrochem Bioenerg 1992; 27(3):405-11.
2. Beneduci A, Chidichimo G, Tripepi S, Perrotte E. Transmission electron microscopy study of the effects produced by wide-band low-power millimeter waves on MCF-7 human breast cancer cells in culture. Anticancer Res 2005; 25(2A):1009-13.
3. Bush LG, Hill DW, Riazi A, Stensaas LJ, Partlow LM, Gandhi OP. Effects of millimeter-wave radiation on monolayer cell cultures. III. A search for frequency-specific athermal biological effects on protein synthesis. Bioelectromagnetics 1981; 2(2):151-9.
4. Chidichimo G, Beneduci A, Nicoletta M, Critelli M, De RR, Tkatchenko Y, Abonante S, Tripepi S, Perrotta E. Selective inhibition of tumoral cells growth by low power millimeter waves. Anticancer Res 2002; 22(3):1681-8.
5. Gapeyev AB, Aripovsky AV, Kulagina TP. Modifying effects of low-intensity extremely high-frequency electromagnetic radiation on content and composition of fatty acids in thymus of mice exposed to X-rays. Int J Radiat Biol 2015; 91(3):277-85.
6. Grundler W, Keilmann F. 1977. Nonthermal effects of millimeter microwaves on yeast growth. Z Naturforsch 33(1-2):15-22.
7. Kalantaryan VP, Vardevanyan PO, Babayan YS, Gevorgyan ES, Hakobyan SN, Antonyan AP. 2010. Influence of low intensity coherent electromagnetic millimeter radiation (EMR) on aqua solution of DNA. Progress in Electromag Res 13:1-9.
8. Khizhnyak EP, Ziskin MC. 1996. Temperature oscillations in liquid media caused by continuous (nonmodulated) millimeter wavelength electromagnetic irradiation. Bioelectromagnetics 17(3):223-9.
9. Manikowska E, Luciani JM, Servantie B, Czerski P, Obrenovitch J, Stahl A. 1979. Effects of 9.4 GHz microwave exposure on meiosis in mice. Experientia 35(3):388-90.
10. Romanenko S, Siegel PH, Pikov V. 2013. Microdosimetry and physiological effects of millimeter wave irradiation in isolated neural ganglion preparation. IEEE 2013 International kharkov symposium on physics and engineering of microwaves, millimeter and submillimeter waves. IEEE 13:512-516.
11. Shckorbatov YG, Pasiuga VN, Kolchigin NN, Grabina VA, Batrakov DO, Kalashnikov VV, Ivanchenko DD, Bykov VN. 2009. The influence of differently polarised microwave radiation on chromatin in human cells. Int J Radiat Biol 85(4):322-329.
12. Smolyanskaya AZ, Vilenskaya RL. 1974. Effects of millimeter-band electromagnetic radiation on the functional activity of certain genetic elements of bacterial cells. Soviet Phys USPEKHI 16(4): 571.
13. Stensaas LJ, Partlow LM, Bush LG, Iversen PL, Hill DW, Hagmann MJ, Gandhi OP. 1981. Effects of millimeter-wave radiation on monolayer cell cultures. II. Scanning and transmission electron microscopy. Bioelectromagnetics 2(2):141-50.
14. Subbotina TI, Tereshkina OV, Khadartsev AA, Yashin AA. 2006. Effect of low-intensity extremely high frequency radiation on reproductive function in Wistar rats. Bull Exp Biol Med 142(2):189-90.

15. Torgomyan H, Kalantaryan V, Trchounian A. 2011. Low intensity electromagnetic irradiation with 70.6 and 73 GHz frequencies affects Escherichia coli growth and changes water properties. Cell Biochem Biophys 60(3):275-81.
16. Webb SJ, Booth AD. 1969. Absorption of microwaves by microorganisms. Nature 222(5199):1199-200.
17. Webb SJ, Booth AD. 1971. Microwave absorption by normal and tumor cells. Science 1;174(4004):72-4.
18. Webb SJ, Dodds DD. 1968. Inhibition of bacterial cell growth by 136 GC microwaves. Nature 218(5139):374-5.
19. Zhadobov M, Sauleau R, Vié V, Himdi M, Le Coq L, Thouroude D. 2006. Interactions between 60-GHz millimeter waves and artificial biological membranes: dependence on radiation parameters. IEEE Trans on micro theory and tech 54(6):2534-42.
